# Implementation of a home blood pressure monitoring program for the management of hypertensive disorders of pregnancy, an observational study in British Columbia, Canada

**DOI:** 10.1177/1753495X231172050

**Published:** 2023-05-07

**Authors:** Karen C Tran, Sabina Freiman, Tessa Chaworth-Musters, Susan Purkiss, Colleen Foster, Nadia A Khan, Wee Shian Chan

**Affiliations:** 1 Division of General Internal Medicine, Department of Medicine, 12358 University of British Columbia, Vancouver, Canada; 2 Center for Health Evaluation and Outcome Sciences, Vancouver, British Columbia, Canada; 3 Internal Medicine Residency Training Program, 12358 University of British Columbia, Vancouver, Canada

**Keywords:** Pre-eclampsia, hypertension, pregnancy-induced, pregnancy, complications, cardiovascular

## Abstract

**Background:**

COVID-19 pandemic has influenced health care delivery. We conducted an observational study to understand how obstetric medicine (ObM) physicians utilized home blood pressure monitoring (HBPM) to manage hypertension in pregnancy.

**Methods:**

Pregnant participants with risk factors or diagnosis of hypertensive disorders of pregnancy (HDP) were enrolled, May 2020–December 2021, and provided with validated home blood pressure (BP) monitor. ObM physicians completed questionnaires to elicit how home BP readings were interpreted to manage HDP.

**Results:**

We enrolled 103 people: 44 antepartum patients (33.5 ± 5 years, gestational age of 24 ± 5 weeks); 59 postpartum patients (35 ± 6 years, enrolled 6 ± 4 days post-partum). ObM physicians used range of home BP readings (70%) for management of HDP.

**Conclusions:**

HBPM to manage HDP is acceptable and can be used to manage hypertension during pregnancy. Further studies are needed to assess the generalizability of our findings and the safety of HBPM reliance alone in management of HDP.

## Introduction

Globally, hypertensive disorders of pregnancy (HDP) including preeclampsia are a leading cause of maternal and infant morbidity and mortality.^1^ International guidelines recommend frequent blood pressure (BP) monitoring in women at increased risk of developing HDP.^2^

Hypertension clinical practice guidelines strongly endorse home blood pressure monitoring (HBPM) over office BP readings for BP monitoring for non-pregnant adults^3–6^ given better predictability for future CV events, reduced risk of white coat hypertension, increased patient engagement and medication adherence with HBPM.^7^ HBPM is also being increasingly advised for pregnant women who are at risk for HDP.^8,9^ Currently, there are no clear consensus on out-of-office BP threshold (home and ambulatory BP) for diagnosis of hypertension, initiation of anti-hypertensive medication and target in pregnancy, both ante-partum and post-partum. Furthermore, variation exists amongst different international guidelines.^7,10,11^ Many physicians do not advocate home BP devices validated in pregnant woman,^12^ do not recommend standardized monitoring schedules,^9^ nor provide patient education on interpretation of home readings and when to seek help for abnormal BP values.^13^

Use of HBPM to manage HDP is unclear as it has not been well studied in pregnancy. As a result, there is large variation on how to use HBPM for management of HDP in clinical practice. The COVID-19 pandemic accelerated the use of HBPM in clinical practice due to limited in-person appointments and provided us an opportunity to evaluate the use of HBPM for management of HDP in the ante- and post-partum period.

We conducted a prospective observational study to assess utilization of HBPM readings for BP management in pregnancy by obstetric medicine (ObM) care providers. Using a validated home BP device and standardized patient education and monitoring schedule, we: (i) explored how ObM physicians interpret and utilize HBPM in the management of pregnant patients with HDP, and (ii) determined acceptability and feasibility of HBPM amongst pregnant woman with HDP.

## Methods

### Study design

The study was conducted as a quality improvement study on the use of HBPM in pregnant women in the ante-partum and post-partum period at British Columbia Women's Hospital OBM clinic in Vancouver, Canada. Ethics approval was waived from the University of British Columbia Research Ethics Board (H19-03400). Informed consent was obtained from all patients.

### Study population

Pregnant people greater than 20 weeks' gestational age with risk factors for preeclampsia, diagnosis of HDP, or post-partum preeclampsia were recruited. Inclusion criteria included those age ≥18 years old; English speaking; able to provide informed consent; greater than 20 weeks’ gestation; diagnosis of hypertensive disorder of pregnancy (chronic hypertension (SBP > 140 mm Hg or DBP > 90 mm Hg before 20 weeks' gestation), gestational hypertension (SBP > 140 mm Hg or DBP > 90 mm Hg after 20 weeks' gestation), or isolated office SBP or DBP reading greater than 140 or 90 mm Hg, respectively), with at least one additional risk factors for developing preeclampsia (i.e.: previous history of preeclampsia, anti-phospholipid antibody syndrome, pre-existing renal disease or booking proteinuria, preexisting diabetes, age ≥40 years, family history of preeclampsia (mother or sister) or early onset cardiovascular disease, multiple pregnancy, overweight/obesity, first pregnancy, pregnancy with a new partner, assisted reproductive technology, or inter-pregnancy interval ≥10 years).^14^ Participants were excluded if they were unable to perform HBPM or attend ObM clinic visits.

### Procedures

#### Home BP monitoring protocol

Participants were provided with an automated home BP monitor (Microlife WatchBP) validated for pregnancy and preeclampsia.^15^ They were provided with in-person training and written instructions for HBPM by the prescribing physician and a paper diary to enter home BP measurements.

Participants were asked to monitor their BP twice in the morning and evening for 1 week prior to each clinical visit and to manually enter BP measurements into a paper diary as these values were used to calculate home BP average. Participants could measure home BP outside of these intervals, but these values were not used to calculate home BP average.^3^ Participants were provided with educational material on BP parameters and symptoms of preeclampsia for which to seek mediation attention (Supplemental material). Home BP thresholds for diagnosis of hypertension and targets were defined as a 6-day average of less than 135/85 mm Hg based on previous systematic review and current guidelines.^16^

#### Clinical management of blood pressure

At each clinic, participants submitted their home BP logs to the ObM physicians by email. HBPM were averaged by discarding the first day's readings, and averaging day 2 to 7 readings.^3^ Participants also would have their BP measured by clinic nurse using standard office BP techniques^17^ if an in-person clinical visit was conducted. Average home BP, raw home BPs, and average office BP readings were available to the ObM physician; following each patient contact, the physician completed a short survey to understand which modality aided the most in medical management and what interpretation of home reading they used (Supplemental material). Physicians arranged follow-up visits as clinically indicated. Physicians who participated in this study included ObM staff, residents, and fellows.

#### Participant survey

Our team jointly developed the questionnaire that was then piloted by ObM physicians prior to dissemination to ensure face validity and understandability of questions. An online open survey using University of British Columbia Qualtrics Online Software was sent to all participants 4 weeks after enrolling in the study (June 2020 to January 2022) to assess (1) understanding of instructions, (2) barriers to performing HBPM, (3) preference of home versus office BP measurement (Supplemental Appendix). Three email reminders were sent at 1, 2, and 4 weeks after the invitation to improve response rate.

### Outcomes

The primary outcome was to describe how ObM physicians used and interpreted home BP measurements to manage clinical care. The secondary outcome was the proportion of patient visits where ObM physicians utilized 7-day average home BP values versus home BP range versus last 3-day values to make clinical decisions on HDP management.

HBPM compliance was assessed by calculating the number of completed home BP readings in one week prior to clinic visit divided by the expected number (the number of home BP readings requested). A priori data was stratified by time point that patients were enrolled (ante-partum vs post-partum). Other clinical data were extracted from the clinical record. Participant acceptability of HDP was assessed by survey response data.

### Statistical analysis

Only participants with a completed physician assessment survey and/or patient experience survey were included in the analysis. Missing data was excluded in the analysis with proportions described under the resulting group size (*n*). Descriptive statistics were used. Continuous variables were presented as either mean (standard deviation) or median (interquartile range (IQR)), and categorical variables were presented as proportions.

## Results

**Figure 1.**
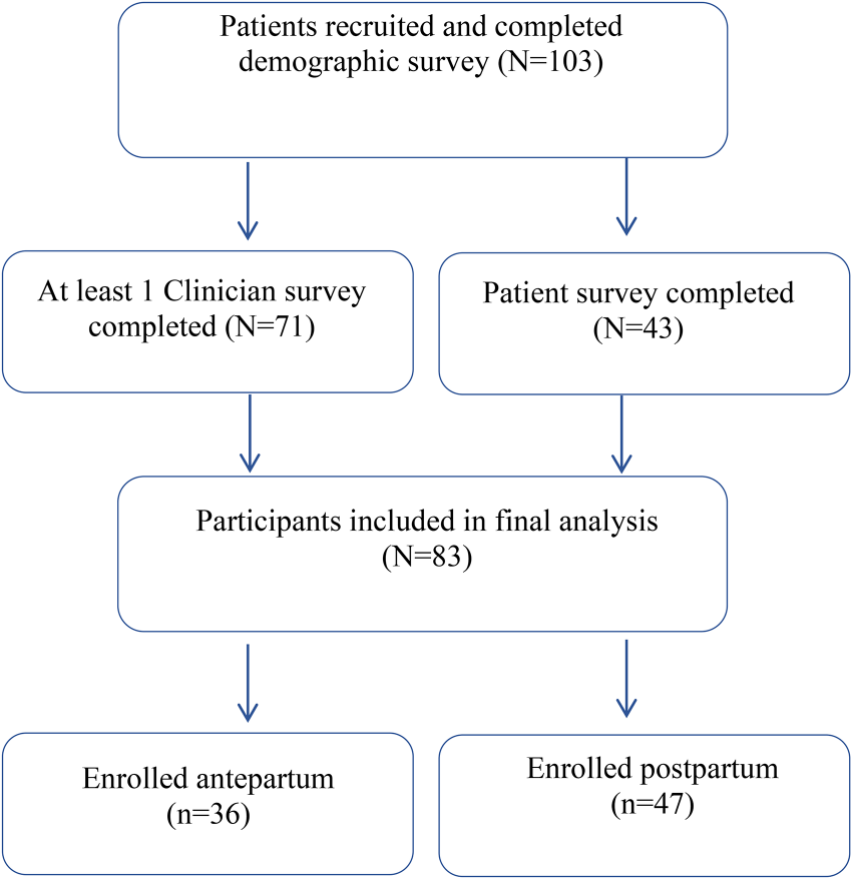
Flow chart describing participation in cohort.

In total, 103 women were enrolled into the study, of which, 44 (43%) were antepartum and 59 (57%) were postpartum from May 2020 to December 2021. Figure 1 shows a flow chart describing participation in our cohort. Of the 103 enrolled participants with a completed background survey, 83 patients had either a completed ObM physician survey or patient survey and were included in the final analysis. Of these 83 participants, 31 had at least one ObM physician survey and the patient survey completed, 40 had only ObM physician surveys completed but no patient survey, and 12 had completed the patient surveys, but no ObM physician surveys were completed. Of the 71 patients with completed ObM physician surveys, they had one to five subsequent visits with surveys completed, accounting for a total of 141 unique clinical visits.

Of these, 60 visits occurred in the antepartum period, and 81 in the postpartum period, with some participants (*n* = 4) studied in both antepartum and postpartum period. Forty-three participants (response rate 41%) completed the qualitative survey (antepartum (40%) and postpartum (60%)).

### Baseline characteristics

 Table 1 summarizes the baseline characteristics. The two groups were comparable in age (35 ± 5 years in the antepartum group and 35 ± 6 years in the postpartum group). Mean gestation age at enrolment was 24 ± 5 weeks and 6 ± 5 days in the antepartum and postpartum, respectively. Of note, women had similar levels of higher education and the population was ethnically diverse. In the antepartum group, 59% were enrolled because they had risk factors for hypertension, whereas in the postpartum, 86% were recruited because of pre-eclampsia. The most common pre-existing comorbidities were hypertension (23%) and diabetes (20%). The most common anti-hypertensive medication prescribed was labetalol (81%) and nifedipine extended release (44%).

**Table 1.** Baseline characteristics of participants in our cohort stratified by ante-partum versus post-partum enrolment.

	*N* (%)		
	Total (*N* = 83)	Antepartum (*N* = 36)	Post-partum (*N* = 47)
Age, median (IQR), y	35.0 (6)	35.5 (6.25)	35.0 (6)
Gestation at entry, mean (SD)		25 (9) weeks	6.2 (4.6) days
Parity	55 (66.3%)	26 (72.2%)	29 (61.7%)
*Race and ethnicity*			
*N*	79	35	44
White	34 (43.0%)	15 (42.9%)	19 (43.2%)
Asian	22 (27.5%)	11 (31.4%)	11 (25.0%)
South Asian	19 (11.4%)	3 (8.6%)	6 (13.6%)
Latin American	5 (6.3%)	2 (5.7%)	3 (6.8%)
Black	4 (4.1%)	3 (8.6%)	1 (2.3%)
Indigenous	1 (1.3%)	1 (2.9%)	0 (0.0%)
Mixed	4 (5.1%)	0 (0.0%)	4 (9.1%)
*N*	79	35	44
Less than high school	1 (1.3%)	1 (2.9%)	0 (0.0%)
High School	7 (8.9%)	4 (11.4%)	3 (6.8%)
Some college, no degree	11 (13.9%)	5 (14.3%)	6 (13.6%)
College	27 (34.2%)	11 (31.4%)	16 (36.4%)
University	15 (19.0%)	4 (11.4%)	11 (25.0%)
Professional qualifications	2 (2.5%)	2 (5.7%)	0 (0.0%)
Masters or PhD	16 (20.3%)	8 (22.9%)	8 (18.2%)
No.	78	33	45
Risk factors for preeclampsia	13 (16.7%)	12 (36.4%)	1 (2.2%)
Chronic hypertension	15 (19.2%)	13 (39.4%)	2 (4.4%)
Preeclampsia	38 (48.7%)	1 (3.0%)	37 (82.2%)
Post-partum hypertension	4 (5.1%)	0 (0.0%)	4 (8.9%)
White coat hypertension	8 (10.3%)	7 (21.2%)	1 (2.2%)

### Clinical management of blood pressure

**Figure 2.**
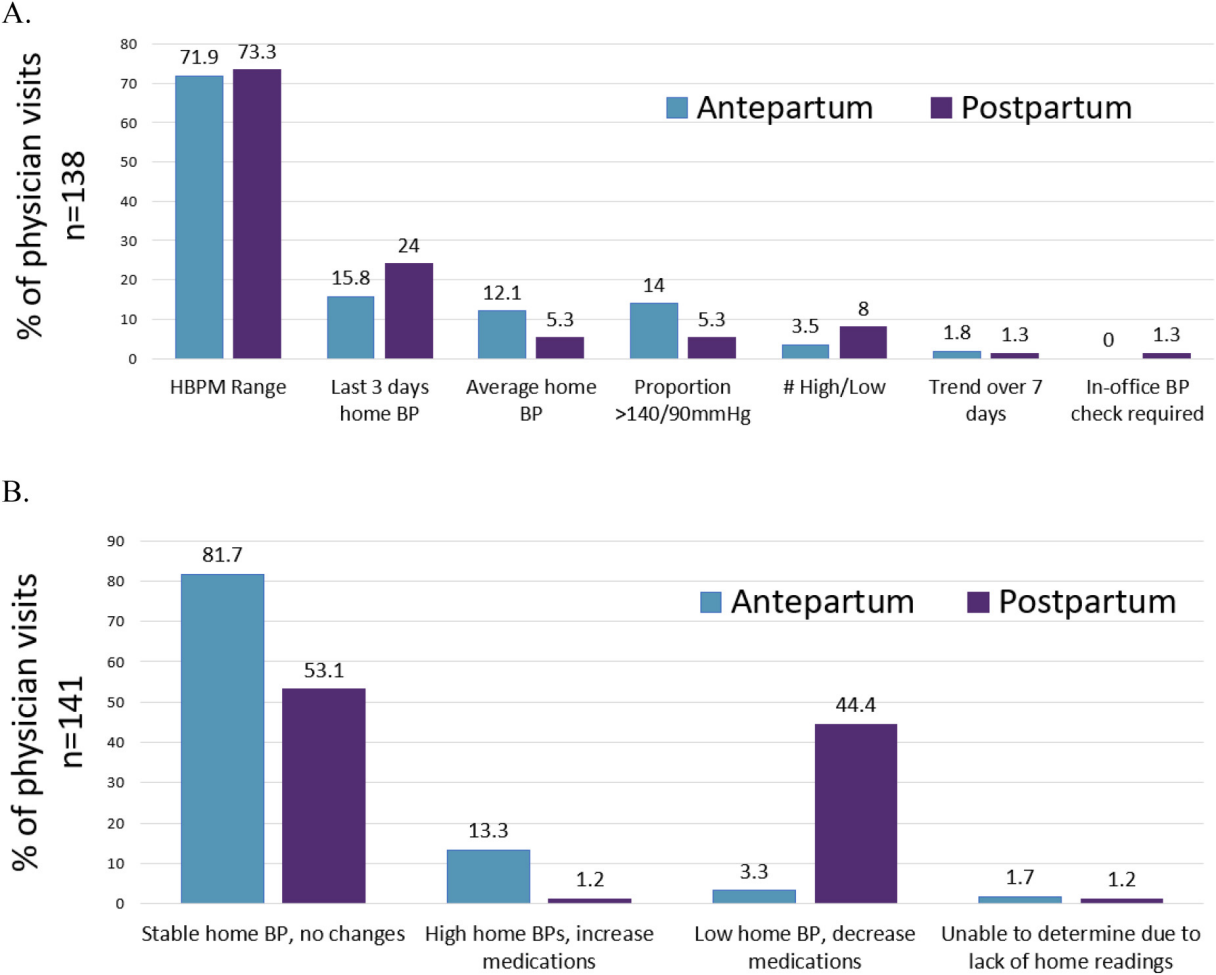
Obstetric medicine physicians (A) preference of home blood pressure metric used to evaluate participant and (B) clinical decision made in antepartum and post-partum period.

Majority of clinical encounters were conducted virtually due to COVID-19 pandemic restrictions at our hospital. In both groups, physicians most commonly used the range of home BP readings to guide their decisions of antepartum (72%) and postpartum (73%) visits. In the antepartum group, physicians used home BP average (21%) and the last three days of home BP readings (16%), compared to postpartum visits where they used the last 3 days of BP readings (24%) more frequently than home BP average (5.3%). Approximately 18% of clinical visits, physicians used multiple metrics to guide hypertension management. Only in one clinical visit (0.7% of all patient visits) did a physician reported needing to use an in-office BP check to influence their clinical decision-making (Figure 2).

In the antepartum group, 82% of clinical encounters resulted in no medication adjustments following the physician's assessment. Only 13% of clinical encounters required increase or initiation of an anti-hypertensive medications due to elevated home BP. In contrast, 53% and 44% of postpartum visits resulted in no medication changes, or decrease/cessation of anti-hypertensive mediations, respectively.

Women adhered with BP readings across both groups (median adherence 0.94, IQR 0.57, 1.00).

### Participant survey

**Figure 3.**
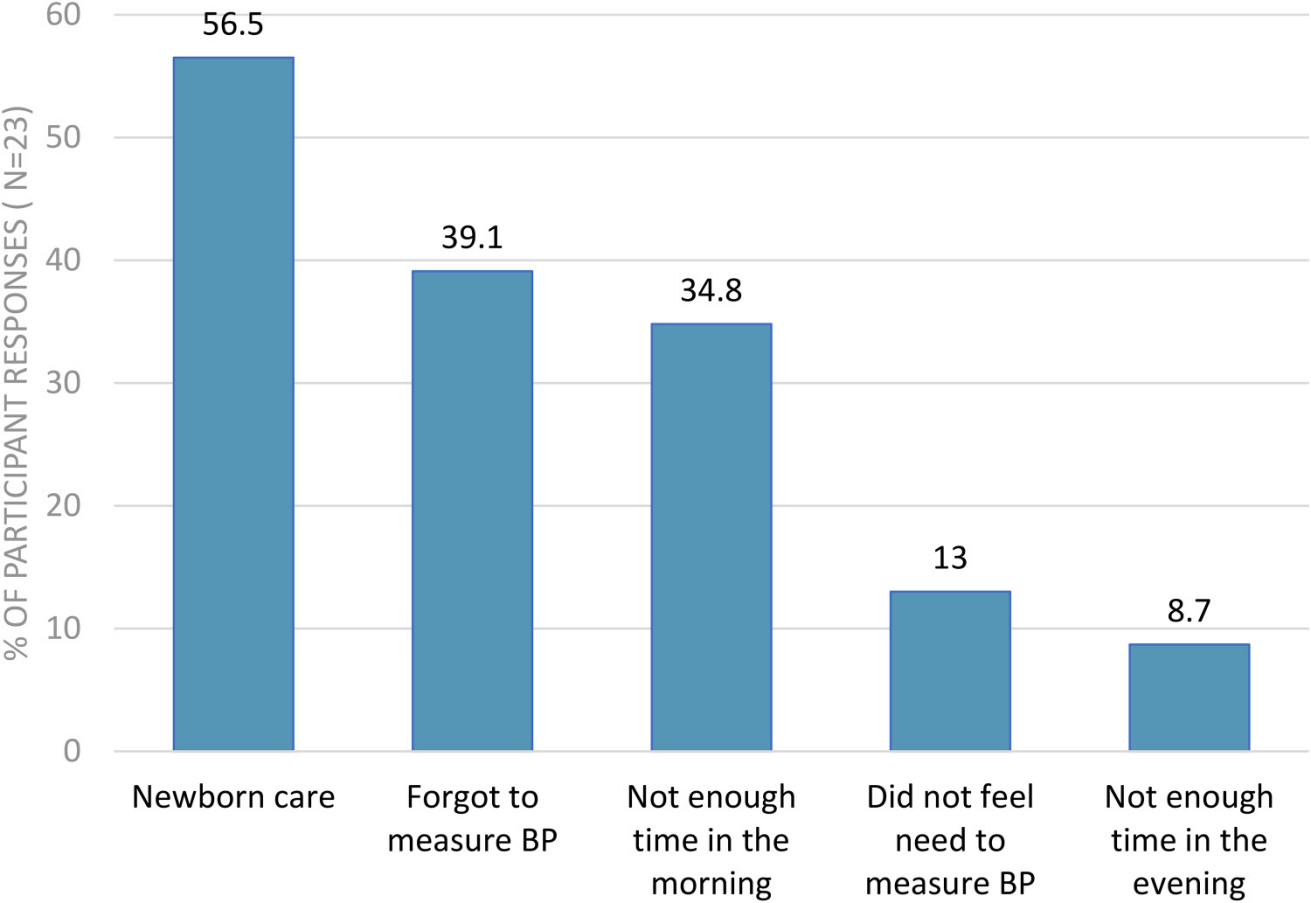
Participant's opinions on barriers to home blood pressure monitoring during pregnancy.

As noted in Table 2, almost all (98%) found HBPM easy to do, and 51% were able to adhere to their monitoring schedule. The most common difficulties with adhering to BP measurement included newborn care (57%), forgetting to check (39%), and lack of time in the mornings (35%) (Figure 3). Almost 48% of participants were reassured by home measurements, but 21% found HBPM increased their anxiety. Participants preferred to have both home and clinic BP measurements (49%), whereas 46% preferred only home BP measurements. In future studies, 62% preferred to email their results; either by diary or spreadsheet, and 38% via a telemonitoring BP app.

**Table 2.** Participants opinions on home blood pressure monitoring in pregnancy.

	*N* (%) *N* = 43
HBPM easy to do	42 (97.7%)
Able to strictly adhere to your HBPM schedule	22 (51.2%)
Difficulties with adhering to home monitoring schedule	*N* = 23
Newborn care	13 (56.5%)
Forgot to measure my blood pressure	9 (39.1%)
Not enough time in the morning	8 (34.8%)
I was feeling well and did not feel I needed to measure my blood pressure	3 (13.0%)
Not enough time in the evening	2 (8.7)
Effect of home blood pressure monitoring on pregnancy	*N* = 42
Reassured me	20 (47.6%)
No effect	13 (31.0%)
Increased my anxiety	9 (21.4%)
Preference on home BP transmission to physician in the future	*N* = 34
E-mail paper diary	15 (44.1%)
App	13 (38.2%)
E-mail spreadsheet	6 (17.6%)
Preference of BP monitoring in the future	*N* = 41
Home	19 (46.3%)
Clinic	2 (4.9%)
Home and clinic	20 (48.8%)

## Discussion

This is the first study to our knowledge that evaluated how ObM physicians used the HBPM readings to make clinical decisions in the real world. Our study highlights that (1) ObM physicians were able to manage HDP using home BPs only, (2) the range of home BP values were commonly utilized and (3) HBPM was acceptable to pregnant women.

Our study demonstrated that ObM physicians were able to manage hypertension using home BP alone, as physician-patient contact were often not done in-person during the COVID-19 pandemic. There was variability in what metric physicians used to interpret HBPM readings: although home BP averages are referenced in most hypertension guidelines for pregnancy,^14^ ObM physicians more commonly relied on the range of HBPM readings and last 3 days of HBPM readings, rather than calculated average home BP to manage hypertension in pregnancy. The reasons for differences in home BP metrics in antepartum and postpartum may be due to follow-up duration. Current clinical practice at our institution is to have patients with post-partum hypertension followed up by ObM 3–7 days post-discharge. Therefore, it is possible that post-partum patients may not have had adequate number of readings to calculate a home BP average. Similarly, clinicians in other studies have relied on home BP trends and ranges, rather than single outlier values.^18^ However, there is lack of guidance on how to incorporate home BP readings into clinical care.

Successful use of HBPM requires integration of HBPM into clinical care and shared decision making between patients and clinicians. Recent RCTs, BUMP1 and BUMP2 did not demonstrate a significant difference in time to diagnosis of hypertension or in mean clinic BP in HBPM compared to usual in-office BP measurements among patients with gestational or chronic hypertension, respectively.^19,20^ Even though women who self-monitor BP noticed higher BPs at home 1 month prior to clinical diagnosis of hypertension, it is suggested that clinicians underutilize home readings for management of HDP.^19^ It is hypothesized that clinicians may have felt uncomfortable adjusting and initiating anti-hypertensive medications based on HBPM readings alone, which may have contributed to no differences in daily dispended dose of anti-hypertensive medications.^20^ Furthermore, it is unclear if elevated home BP readings resulted in additional in-person assessments of BP. In our study, less than 1% of clinical encounters had a subsequent in-office assessment of BP to guide management decisions due to COVID-19 pandemic restrictions; otherwise, all decisions were based on interpretations of home BP measurements.

While HBPM was reassuring for many (47.6% of survey respondents), 21.4% reported increased anxiety. Reassuringly, other studies showed no significant differences between home monitoring and usual care with respect to increasing anxiety across the pregnancy period.^19,21^ Previous studies in pregnancy using HBPM demonstrated most women were confident in their ability to self-monitor BPs and provide sense of reassurance and control.^18,21,22^ This suggests that HBPM is acceptable to pregnant women.

Our study was the first to our knowledge to assess how physicians utilize and integrate HBPM to make clinical decisions for women with hypertension in pregnancy, both in the ante-partum and post-partum period. HBPM appears to be a feasible tool which physicians can use to manage pregnant women at risk of HDP. Our study is limited by referral bias, its non-randomized design with no control group, and missing data based on variable physician and patient participation. Our study was not designed to understand the differences in why physicians used different home BP metrics to make clinical decisions for HDP in the ante-partum and post-partum period. These need to be evaluated and validated in future studies. Furthermore, the survey had a low survey response rate that may exclude those that struggled more with HBPM and may be subject to recall bias. Another limitation is the use of closed questions on the patient survey to assess HBPM acceptability and barriers to compliance. Future qualitative studies are needed to understand barriers to HBPM and solutions to overcome them. Finally, we did not systematically correlate our findings to maternal or neonatal outcomes.

Recent large RCTs have shown that HBPM is safe in pregnancy, with no differences in maternal or neonatal outcomes.^19,20^ However, clinical equipoise exists on how to best incorporate home BP readings to make clinical decisions for HDP management, specifically in titrating anti-hypertensive medications. With increasing dependence on virtual appointments in light of the COVID-19 pandemic, there is greater need for exploration of the virtual management of HDP. Future directions would include large scale, multicentre RCT on using home BP measurements to titrate anti-hypertensive medications, integration into clinical care and to assess differences in maternal and neonatal outcomes.

## Conclusions

HBPM has the potential to manage HDP during the ante-partum and post-partum period. In light of the COVID-19 pandemic and increasing demand for virtual healthcare visits, future multicentre randomized controlled studies are need to assess the effectiveness, safety and acceptability of HBPM on management of HDP.

## Supplemental Material

sj-pdf-1-obm-10.1177_1753495X231172050 - Supplemental material for Implementation of a home blood pressure monitoring program for the management of hypertensive disorders of pregnancy, an observational study in British Columbia, Canada Supplemental material, sj-pdf-1-obm-10.1177_1753495X231172050 for Implementation of a home blood pressure monitoring program for the management of hypertensive disorders of pregnancy, an observational study in British Columbia, Canada by Karen C Tran, Sabina Freiman, Tessa Chaworth-Musters, Susan Purkiss, Colleen Foster, Nadia A Khan and Wee Shian Chan in Obstetric Medicine

sj-docx-2-obm-10.1177_1753495X231172050 - Supplemental material for Implementation of a home blood pressure monitoring program for the management of hypertensive disorders of pregnancy, an observational study in British Columbia, Canada Supplemental material, sj-docx-2-obm-10.1177_1753495X231172050 for Implementation of a home blood pressure monitoring program for the management of hypertensive disorders of pregnancy, an observational study in British Columbia, Canada by Karen C Tran, Sabina Freiman, Tessa Chaworth-Musters, Susan Purkiss, Colleen Foster, Nadia A Khan and Wee Shian Chan in Obstetric Medicine
