# Supplementary material for: Implementation of a home blood pressure monitoring program for the management of hypertensive disorders of pregnancy, an observational study in British Columbia, Canada
Source: Obstet Med. 2023 May 7;17(1):22–7. doi: 10.1177/1753495X231172050 (PMC11037197; doi:10.1177/1753495X231172050)
Supplement: sj-pdf-1-obm-10.1177_1753495X231172050 - Supplemental material for Implementation of a home blood pressure monitoring program for the management of hypertensive disorders of pregnancy, an observational study in British Columbia, Canada [file sj-pdf-1-obm-10.1177_1753495X231172050.pdf]

BC WOMEN'S HOSPITAL+ HEALTH CENTRE
An agency of the Provincial Health Services Authority

How to Measure Blood Pressure at Home

1. Take readings twice in the morning (within two hours of waking up) and two readings before going to bed (four readings in total each day). Take readings 1-2 minutes apart.

2. Make sure you are seated, with your back and legs supported.

3. Your arm that has the blood pressure cuff on should be at heart level.

4. Readings are most accurate when you are alone and rested for 5 minutes, without any distractions including reading emails, checking your phone, baby care etc.

5. Try to avoid caffeine or smoking 1 hour before taking your BP.

6. Try to ensure an empty bladder prior to measuring your BP.

7. You will be instructed by your physician regarding the number of days to take your BP before your next visit.

8. Please record your BP on either the home BP diary or EXCEL spreadsheet that is provided.

9. If your blood pressure is greater than 150/100 (based on 2 readings 15 minutes apart) or you have symptoms of preeclampsia (headache, vision changes, chest pain, shortness of breath or liver pain), contact the Obstetrical Medicine Clinic between 830am-430pm at 604-875-2160. After 430 pm and before 830 am: Call BC Women's Hospital (604-875-2000) and ask to speak with the on-call physician for Obstetrical Medicine

10. Please email your home BP diary to OBIMdocs@cw.bc.ca before your next appointment with your Obstetrical Medicine Specialist

My Blood Pressure Diary (Systolic BP/Diastolic BP)

Please measure your BP for 7 days/daily/ or ___________ before next appointment
Instructions provided by _________________MD

	**MORNING BP**			**BEDTIME BP**		
	Reading #1	Reading #2	Comments	Reading #1	Reading #2	**Comments**
Example	146 /78	152 /74	Before meds	110/78	116/82	Upset
**Day 1**	/	/		/	/	
**Day 2**	/	/		/	/	
**Day 3**	/	/		/	/	
**Day 4**	/	/		/	/	
**Day 5**	/	/		/	/	
**Day 6**	/	/		/	/	
**Day 7**	/	/		/	/	

Calculate the Average Systolic and Diastolic Pressures

Average Systolic = Add up all systolic pressures from Days 2-7 (throw out day 1 readings) and divide by 24

Average Diastolic = Add up all diastolic pressures from Days 2-7 and divide by 24

MY AVERAGE BP: _________________/_____________________
