# Supplementary material for: Implementation of a home blood pressure monitoring program for the management of hypertensive disorders of pregnancy, an observational study in British Columbia, Canada
Source: Obstet Med. 2023 May 7;17(1):22–7. doi: 10.1177/1753495X231172050 (PMC11037197; doi:10.1177/1753495X231172050)
Supplement: sj-docx-2-obm-10.1177_1753495X231172050 - Supplemental material for Implementation of a home blood pressure monitoring program for the management of hypertensive disorders of pregnancy, an observational study in British Columbia, Canada [file sj-docx-2-obm-10.1177_1753495X231172050.docx]

**Patient Online Survey**

1. Were the written instructions provided to you on how to measure blood pressure at home clear and easy to understand?

- Yes
- No

1. Was measuring blood pressure at home easy to do?

- Yes
- No

1. If you answered No to question 2, why was home blood pressure monitoring difficult? (Please select as many answers that apply)

- The instructions were not clear
- The cuff was uncomfortable
- The cuff was too big or too small
- The blood pressure machine did not work properly
- There were error messages with the device
- The battery did not last
- I didn’t have enough time to do it
- There were too many distractions at home to do it
- I couldn’t remember when to measure my blood pressure
- Other reason:

1. Were you able to strictly adhere to your blood pressure monitoring schedule?

- Absolutely Yes
- Sometimes No

1. If you answered Sometimes No to Question 4, please indicate difficulties with the monitoring schedule (Select as many answers that apply)

- I didn’t have enough time in the mornings
- I didn’t have enough time in the evenings
- I forgot when to measure my blood pressure
- It took too long to measure my blood pressure
- Not a priority
- There were too many blood pressure measurements required
- I felt well and didn’t feel that I needed to measure my blood pressure
- Other reason:

1. How do you prefer to transmit your home blood pressure readings to your physician?

- Email paper home BP diary
- Email Excel spreadsheet
- App in the future is preferred

1. How did home blood pressure monitoring affect your pregnancy experience?

- No affect
- Increased my anxiety
- Reassured me

1. Do you prefer having your blood pressure measured at home or at the clinic?

- Home
- Clinic
- Both
- Neither

1. How much would you be willing to pay for an accurate home blood pressure machine?

- I am unable to pay for a home BP machine
- <$50
- <$100
- <$200

1. Would you be interested in participating in an in-person interview to discuss your experience with home blood pressure monitoring?

- Yes
- No
